# The* Ginkgo biloba* Extract Reverses the Renal Effects of Titanium Dioxide Nanoparticles in Adult Male Rats

**DOI:** 10.1155/2016/5781579

**Published:** 2016-03-03

**Authors:** Carlos Enrique Escárcega-González, Irma Guadalupe Reynoso-Andeola, Fernando Jaramillo-Juárez, Haydée Martínez-Ruvalcaba, Francisco A. Posadas del Rio

**Affiliations:** ^1^Departamento de Fisiología y Farmacología, Centro de Ciencias Básicas, Universidad Autónoma de Aguascalientes, 20131 Aguascalientes, AGS, Mexico; ^2^Departamento de Microbiología, Centro de Ciencias Básicas, Universidad Autónoma de Aguascalientes, 20131 Aguascalientes, AGS, Mexico

## Abstract

The* Ginkgo biloba* extract (GbE) is a commercial product used as a nutraceutic herbal remedy in Europe and US. It contains 27% of the polyphenols isorhamnetin, kaempferol, and quercetin, as antioxidants. We used male adult Wistar rats (200–300 g), divided into four groups: control group (treated with 5.0 mg/kg of sodium chloride, intravenous), titanium dioxide nanoparticles (TiO_2_-NPs) group (5.0 mg/kg, intravenous), GbE group (10 mg/kg, intraperitoneal), and GbE + TiO_2_-NPs group (treated 24 h before with 10 mg/kg of GbE, intraperitoneal), followed, 24 h later, by 5.0 mg/kg of TiO_2_-NPs intravenously. The statistical analysis was performed using Student's *t*-test for grouped data with ANOVA posttest. The GbE protected renal cells against the effects of TiO_2_-NPs because it reversed the increased activity of *γ*-glutamyltranspeptidase and the enzymatic activity of dipeptidylaminopeptidase IV at all times tested (0–5, 5–24, 24–48, and 48–72 h). Also it reversed the glucosuria, hypernatriuria, and urine osmolarity at three times tested (5–24, 24–48, and 48–72). Thus, we conclude that GbE has a beneficial activity in the cytoplasmic membranes of brush border cells on the renal tubules, against the adverse effects that can be produced by some xenobiotics in this case the TiO_2_-NPs, in experimental rats.

## 1. Introduction

The main function of kidneys, in mammals, is the excretion of metabolic end products from the body and the regulation of extracellular fluid volume and electrolyte composition [1]. Their high blood flow, combined with their ability to concentrate solutes, exposes them to high concentration of xenobiotics present in the systemic circulation. Because of the rich blood supply of the kidneys, in relation to their mass, this organ is particularly liable to damage by toxic substances.

Most living organisms are exposed to nanoparticles (NPs) through the gastrointestinal tract, the lungs, and the skin [2–4]. Moreover, titanium dioxide nanoparticles (TiO_2_-NPs) affect kidney cells* in vitro* [5–7] and* in vivo* [8].

Once into the body, the NPs can interact with cell structures like the plasmatic membrane and cause disruption of it. Some of the biological effects of TiO_2_-NPs in nanomedicine, after intravenous injection, deliver TiO_2_-NPs into the human body: they induce pathological lesions in liver, spleen, kidneys, and brain [9]. Also, the intravenous administration of TiO_2_-NPs (5 mg/kg) in rats causes an accumulation of nanoparticles in the kidneys with the highest burden on a day 1 after exposure and remains until day 14 [10]. Furthermore, after a single oral administration (5 g/kg) in mice, these nanoparticles change some serum biochemical parameters (alanine aminotransferase (ALT), aspartate aminotransferase (AST), LDH, and BUN), and pathology of the kidneys indicated that renal injury was induced after exposure [11].

One of the first ultrastructural changes seen after treatment with many nephrotoxins is sloughing of proximal tubule brush borders [12]. The enzymes *γ*-glutamyltranspeptidase (*γ*-GTP) and dipeptidylaminopeptidase IV (DAP-IV) are predominantly located on the apical membrane (brush border) of proximal cells [13, 14]. Thus, xenobiotics (in this case TiO_2_-NPs) can produce release of *γ*-GTP and DAP-IV from their site in the brush border membrane of renal tubules, causing their urinary excretion (enzymuria). Moreover, along the nephron, the reabsorption and secretion of solutes (sodium and glucose) are done by different cotransporters such as sodium-glucose sodium-phosphate, sodium-amino, Na-K-2Cl, and Na-Cl. Therefore, the damage of the cytoplasmic membrane would disrupt the function of these cotransporters. These transporters are the major participants in urine osmolarity [15].

The* Ginkgo biloba* extract (GbE) is a commercial product, considered as nutraceutic [16] with possible beneficial effects on human health [17–23]. The GbE contains an average of 27% polyphenols isorhamnetin, kaempferol, and quercetin [24–28] and 6% terpene lactones (terpenoid fraction). The terpenoid fraction primarily contains ginkgolides A, B, C, J, and M, as well as bilobalide. Its purported biological effects include free radical scavenging, antiapoptotic, anti-inflammatory, and antioxidative activities [29]. The GbE is used in many applications such as the treatment of dementia, cerebral insufficiency, or related cognitive decline [30]. The possible mechanisms implied in the neuroprotective effect are modulation of ion homeostasis, glucocorticoid levels, and synthesis of growth factors [31]. In recent clinical and experimental experiments, GbE has been reported to be effective against ischemic brain injury [32, 33], cerebral (or cerebrovascular) insufficiency [34], cognitive speed [35], dementia and Alzheimer's disease [36], peripheral vascular disease such as arterial occlusive disease [37], and aging damage [38]. In the case of renal cells, the GbE has renoprotective effect against cisplatin-induced nephrotoxicity [29]. In other study, changes in blood urea, serum creatinine, and creatinine clearance induced by gentamicin were significantly prevented by* Ginkgo biloba* extract [39]. Furthermore, the GbE diminishes adriamycin-induced proteinuria and hyperlipidaemic nephrotoxicity in rats [40].

Our hypothesis was that pretreatment with GbE as a single dose (10 mg/kg of body weight), administered intraperitoneally, would reverse the renal effects of the intravenous administration of a single dose of TiO_2_-NPs, in the kidneys of adult male rats.

## 2. Materials and Methods

### 2.1. Animals and Reagents

Male adult Wistar rats (200–300 g) were used and maintained in stainless steel cages with a 12 h light/dark regime. The rats were handled according to the* Guiding Principles in the Use of Animals in Toxicology*.

*Ginkgo biloba* extract was from Vasodil®, NYCOMED, México, titanium dioxide nanoparticles were from Anatase, Sigma Aldrich, St. Louis, MO, USA, and *γ*-glutamyl p-nitroanilide and gly-pro p-nitroanilide were from Sigma Aldrich, St. Louis, MO, USA.

### 2.2. Experimental Design

Treatment was as follows. The rats were divided into four groups: control group (treated with 5.0 mg/kg of sodium chloride aqueous solution, intravenously or i.v.), titanium dioxide group (5.0 mg/kg of TiO_2_, i.v.),* Ginkgo biloba* group (10 mg/kg, intraperitoneal or i.p.), and* Ginkgo biloba* + titanium dioxide group (treated 24 h before with 10 mg/kg of* Ginkgo biloba* extract, i.p.), followed, 24 h later, by 5.0 mg/kg of TiO_2_, i.p.

The rats were kept with food and water* ad libitum* and at room temperature (24 ± 1°C). The urine was continuously collected, in vessels attached to the metabolic cages, from 0 to 5 h, from 5 to 24 h, from 24 to 48 h, and from 48 to 72 h.

Biochemical assays were as follows. The specific activity of *γ*-glutamyltranspeptidase was determined in 50 mM Tris-Cl, pH 9.0, 10 mM MgCl_2_, with 20 mM glycylglycine and *γ*-glutamyl p-nitroanilide as substrate, in a spectrophotometer at 405 nm [41]. The specific activity of dipeptidylaminopeptidase-IV was assayed in 50 mM Tris-Cl, pH 8.0, with gly-pro p-nitroanilide as substrate, also at 405 nm [42] in a spectrophotometer. The enzymatic activities were carried out at room temperature (25 ± 1°C), in 0.5 mL final incubation volume. The initial enzymatic rates were calculated from continuous recording, usually in duplicates, in a Varian UV/VIS spectrophotometer (Varian DMS 80).

Protein was measured with the Folin phenol reagent using bovine serum albumin as standard [43]. We also measured in urine the volume, the concentration of creatinine [44], the pH in a pH meter, the osmolality in a microosmometer (Osmette, Precision Systems Model 5004), and the concentration of sodium in a flame photometer (Corning M410).

### 2.3. Statistical Analysis

We calculated the significance of the differences between group means with the two-tailed Student's *t*-test for grouped data with ANOVA posttest of the urinary parameters, using the software Prism 4 (GraphPad Software Inc.); graphs were produced using Slide Write Plus version 4.0 for Windows (Advanced Graphics Software Inc.).

## 3. Results

The pretreatment with a single and intraperitoneal dose of the* Ginkgo biloba* extract (GbE) reversed the renal effects of a single dose of TiO_2_-NPs (5 mg/kg, intravenous), studied in the urine of adult male rats.

### 3.1. The Effects of GbE on the Renal Effects of Titanium Dioxide (TiO_2_) of the *γ*-Glutamyltranspeptidase Enzymatic Activity in Urine

**Figure 1:**
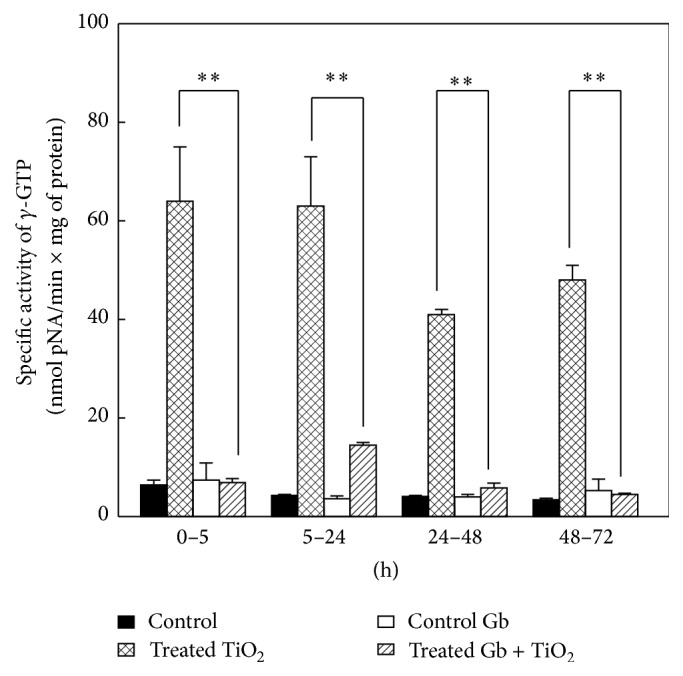
The effects of GbE on the renal effects of titanium dioxide (TiO_2_) of the *γ*-glutamyltranspeptidase enzymatic activity in urine, at different time periods, compared with control, GbE, and TiO_2_-treated rats. The enzymatic activity is shown as nmol p-NA/min × mg of protein. The values represent mean ± SEM, *n* = 6. The significance level is ^*∗∗*^ *P* < 0.01; pNA: p-nitroanilide.

The increased enzymatic activity of *γ*-glutamyltranspeptidase, generated by titanium dioxide, was totally and significantly (*P* < 0.05) reversed with the 24 h pretreatment of GbE, from 64.4 ± 10.7 to 6.9 ± 0.8 (0–5 h), from 63.3 ± 9.6 to 14.5 ± 0.5 (5–24 h), from 40.9 ± 0.6 to 5.8 ± 0.9 (24–48 h), and from 48.3 ± 3.4 to 4.5 ± 0.2 nmol pNA/min × mg of protein (48–72 h), as shown in Figure 1.

### 3.2. The Effects of GbE on the Renal Effects of Titanium Dioxide on the Enzymatic Activity of Urinary Dipeptidylaminopeptidase IV

**Figure 2:**
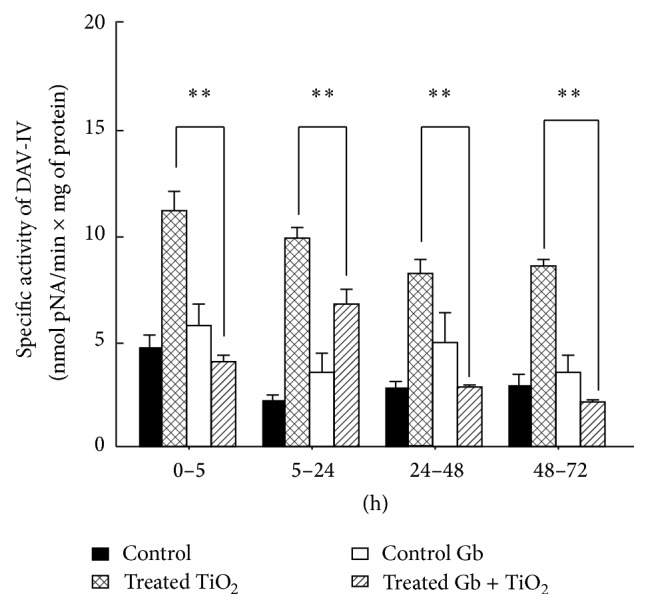
The effects of GbE on the renal effects of titanium dioxide on the enzymatic activity of urinary dipeptidylaminopeptidase IV, at different time periods, compared with control, GbE, and TiO_2_-treated rats. The enzymatic activity is shown as nmol p-NA/min × mg of protein. The values represent mean ± SEM, *n* = 6. The significance level is ^*∗∗*^ *P* < 0.01; pNA: p-nitroanilide.

The GbE partially and significantly (*P* < 0.05) reversed the increase on enzymatic activity of dipeptidylaminopeptidase IV, produced by TiO_2_, from 11.1 ± 0.9 to 4.0 ± 0.3 (0–5 h), from 9.8 ± 0.5 to 6.7 ± 0.7 (5–24 h), from 8.1 ± 0.7 to 2.8 ± 0.1 (24–48 h), and from 8.5 ± 0.3 to 2.1 ± 0.1 nmol pNA/min × mg of protein (48–72 h), as depicted in Figure 2.

### 3.3. The Effects of GbE on the Renal Effects of TiO_2_ of the Glucose Concentration in Urine

**Figure 3:**
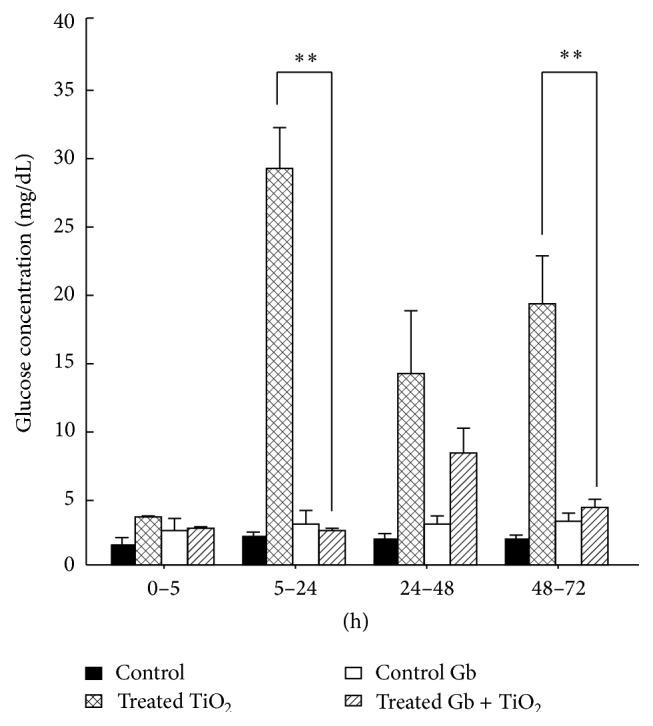
The effects of GbE on the renal effects of TiO_2_ of the glucose concentration in urine, at different time periods, compared with control, GbE, and TiO_2_-treated rats. The glucose concentration is presented as mg/dL. The significance level is ^*∗∗*^ *P* < 0.01.

Figure 3 shows that the GbE decreased significantly (*P* < 0.05) the glucosuria, produced by titanium dioxide, only at 5 to 24 h (29.2 ± 3.2 to 2.5 ± 0.2), and again from 48 to 72 h (19.1 ± 3.5 to 4.8 ± 0.6 mg/dL). The values from 24 to 48 h were not significantly different that control or GbE-treated rats.

### 3.4. The Effects of GbE on the Renal Effects of TiO_2_ of the Urinary Sodium Concentration

**Figure 4:**
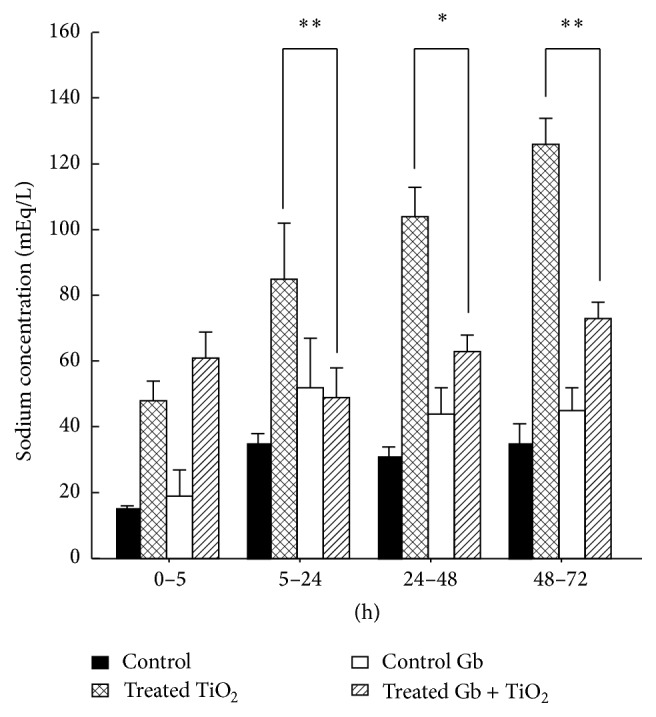
The effects of GbE on the renal effects of TiO_2_ of the urinary sodium concentration, at different time periods, compared with control, GbE, and TiO_2_-treated rats. The sodium concentration is presented as mEquivalents of Na^+^/liter. The significance levels are: ^*∗*^ *P* < 0.05, ^*∗∗*^ *P* < 0.01.

In Figure 4 it is shown that the GbE decreased significantly (*P* < 0.05) the hypernatruria produced by titanium dioxide: from 5 to 24 h (85 ± 17 to 49 ± 9), from 24 to 48 h (104 ± 9 to 63 ± 5), and from 48 to 72 h (126 ± 8 to 73 ± 5 mEq/L).

### 3.5. The Effects of GbE on the Renal Effects of Titanium Dioxide in the Urine Osmolarity

**Figure 5:**
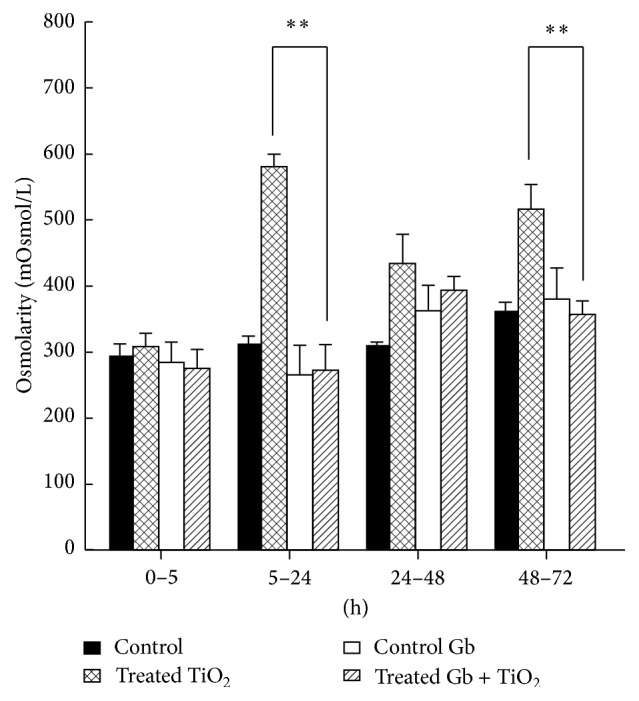
The effects of GbE on the renal effects of titanium dioxide in the urine osmolarity, at different time periods, compared with control, GbE, and TiO_2_-treated rats. The osmolarity is shown as mOsmoles/liter. The significance level is ^*∗∗*^ *P* < 0.01.

The GbE diminished significantly (*P* < 0.05) the urinary hyperosmolarity produced by TiO_2_ only from 5–24 h (580 ± 19 to 272 ± 69) and again from 48 to 72 h (516 ± 7 to 357 ± 20 mOsmol/L), as depicted in Figure 5. The values from 24 to 48 h were not significantly different that control or GbE-treated rats.

### 3.6. The Effects of GbE and Titanium Dioxide on Other Urinary Parameters

Finally, the GbE alone did not significantly modify the concentrations of protein and creatinine, the volume, or the pH of urine, respectively, compared to control or GbE-pretreated rats, as shown in Table 1. Likewise, the GbE did not significantly modify the water and food intakes or the body weight, respectively, also compared to control or GbE-pretreated rats, as depicted in Table 2.

**Figure 6:**
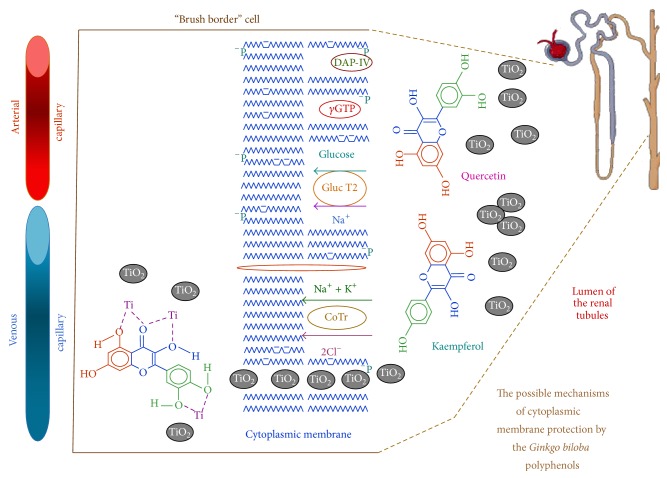
Scheme on the possible mechanisms of the GbE polyphenols for the renoprotection of the cytoplasmic membrane against the effects generated by the TiO_2_ nanoparticles, at the luminal side of the brush border cells, along the renal tubules of rat kidneys. *γ*GT: *γ*-glutamyltranspeptidase, DAP-IV: dipeptidylaminopeptidase IV, CoT: electroneutral Na-K-Cl cotransporter, and Gluc T2: Glucose-Na cotransporter 2.

In Figure 6, we show a scheme about the possible mechanisms of the GbE polyphenols to protect the cytoplasmic membrane from renal effects produced by the titanium dioxide nanoparticles, at the luminal side of the brush border cells, all along the renal tubules of rat kidneys.

## 4. Discussion

The administration of a single and intraperitoneal dose of the* Ginkgo biloba* extract (GbE, 10 mg/kg of body weight) significantly (>0.05) reversed the renal effects of a single and intravenous dose (5 mg/kg) of titanium dioxide (TiO_2_) nanoparticles, studied in the urine of adult male rats. The dose of TiO_2_ that we used is 11.8 times lower than its LD_50_ [45]. TiO_2_-NPs are a fine white powder, often used as pigments or additives for ceramics, paints, paper, plastics, food, sunscreens, and toothpaste [46]. Living organisms are exposed to TiO_2_-NPs and may develop toxic effects. The toxicity of TiO_2_-NPs has mainly been studied* in vitro* [47–50] but with fewer studies* in vivo* [51] and a growing need for* in vivo* research on the effects of nanoparticles [52, 53].

*Ginkgo biloba* is one of the most widely used herbal remedies in Europe and US [54]. It is well known that the GbE contains 27% of the polyphenols isorhamnetin, kaempferol, and quercetin [24–27].

We believe that the renoprotective effects of the GbE, against the effects of TiO_2_ nanoparticles, are mainly due to the higher content and the interaction of the three GbE polyphenols with the cytoplasmic membrane of the brush border cells on the renal tubules and perhaps also to a synergistic effect among them.

The polyphenols interact with model membranes [55–67] and with erythrocyte membranes [68–70]. Some of the previous authors report evidence that this interaction is mainly due to hydrogen bonding between lipid polar head groups of membranes and the hydroxyl groups of polyphenols. This interaction would stabilize the membranes and influence their fluidity by decreasing packing in the hydrophilic region of membrane.

In relation to the specific activity of *γ*-glutamyltranspeptidase (*γ*-GTP) and dipeptidylaminopeptidase IV (DAP-IV), because they are in contact with the lumen of the renal tubules, and due to their location in the brush border membrane of renal proximal tubules [13, 14], they are the most susceptible parameters to the direct adverse effect by TiO_2_-NPs, and thus, they are the most benefited by the GbE at all times tested. This can be explained because the polyphenols exert a screen effect combined with its chelating and antioxidant activity. These properties have been well studied in quercetin [71, 72].

According to the other parameters, the time-related effects of TiO_2_-NPs were detected after the enzymuria, as increased concentration of the urinary glucose (glucosuria) and increased concentration of the urinary sodium (hypernatriuria), along with increased of urine osmolality. Similarly, with its polyphenols, the GbE exerted its beneficial mentioned properties [71, 72], protecting and preventing a dysregulation in the function of nephron cotransporters involved in the regulation of sodium, glucose, and therefore the osmolarity, produced by the TiO_2_-NPs.

Likewise, it is possible that the GbE renoprotection is potentiated by its components as reported* in vivo* for their pharmacokinetics in rats [73] and for the treatment of cancer [74–76]. We also believe that the polyphenols of the GbE participate, as antioxidants [77], in the renoprotective effects against TiO_2_, mainly at later times [78].

We do not rule out the participation of the antioxidant effects of GbE components on the renal effects like oxidative stress, generated by nanoparticles, reported by several authors [55, 79–82].

Finally, we could not find significant differences among the control, the GbE, and the TiO_2_-treated rats for the following urinary parameters: the volume, the concentration of protein, the concentration of creatinine, or the pH. Likewise, the water and food intakes as well as the gain in body weight were not statistically different in control rats, compared with the GbE, and the TiO_2_-treated rats.

## 5. Conclusions

The administration of* Ginkgo biloba* extract (GbE, 10 mg/kg of body weight) by the intraperitoneal route and as a single dose reversed the renal effects of a single and intravenous dose (5 mg/kg) of titanium dioxide (TiO_2_) nanoparticles in male and adult rats, studied in the urine. The GbE recovered the enzymatic activities of *γ*-glutamyltranspeptidase and of dipeptidylaminopeptidase IV. The GbE also reversed the glucosuria, the hypernatruria, and the hyperosmolarity generated by titanium dioxide. These effects appear to be mainly due to the interaction and protection of the GbE polyphenols with the cytoplasmic membrane on renal tubules of the male adult rats. Thus, we conclude that GbE has a beneficial activity in the cytoplasmic membranes of brush border cells on the renal tubules, against the adverse effects that can be produced by some xenobiotics, in this case the TiO_2_-NPs, in experimental rats. Therefore, our research highlights the pharmacological activity of the* Ginkgo biloba* extract, which can be used as an alternative treatment to protect renal cells against the toxicity produced by TiO_2_-NPs.

## Figures and Tables

**Table 1:** The effects of the *Ginkgo biloba* extract (10 mg/kg b.w., intraperitoneal) administered 24 h before titanium dioxide (5 mg/kg b.w., intravenous) on other urinary parameters studied.

Parameter	0–5 h	5–24 h	24–48 h	48–72 h
Protein (mg/mL)				
Control	4.5 ± 0.3	8.8 ± 0.4	8.2 ± 0.2	8.8 ± 0.2
Control Gb	6.8 ± 2.2	7.5 ± 1.8	10.7 ± 0.8	8.2 ± 0.8
Treated TiO_2_	5.3 ± 0.5	5.6 ± 0.3	6.9 ± 0.3	7.8 ± 0.1
Pretreated Gb	6.9 ± 0.3	6.6 ± 1.5	8.3 ± 0.2	7.3 ± 0.2
Total creatinine (mg/dL)				
Control	57 ± 2.1	87 ± 1.7	77 ± 2.7	62 ± 5.1
Control Gb	46 ± 6.9	67 ± 12.8	68 ± 1.9	55 ± 3.9
Treated TiO_2_	47 ± 1.5	77 ± 1.3	83 ± 0.6	84 ± 6.5
Pretreated Gb	47 ± 7.9	56 ± 6.3	63 ± 2.2	50 ± 1.7
Volume (mL)				
Control	4.0 ± 0.5	8.7 ± 0.7	8.7 ± 0.5	13.7 ± 1.4
Control Gb	2.5 ± 0.2	8.0 ± 2	7.0 ± 1.3	7.0 ± 0.9
Treated TiO_2_	3.0 ± 0.15	9.0 ± 1.0	9.0 ± 1.0	9.0 ± 1.0
Pretreated Gb	2.6 ± 0.2	10 ± 2	10 ± 1	11 ± 1
pH				
Control	7.6 ± 0.2	7.4 ± 0.3	7.3 ± 0.3	6.9 ± 0.4
Control Gb	8.0 ± 0.05	7.0 ± 0.5	6.5 ± 0.2	7.8 ± 0.1
Treated TiO_2_	6.5 ± 0.3	6.5 ± 0.3	6.8 ± 0.2	6.3 ± 0.2
Pretreated Gb	7.0 ± 0.3	7.8 ± 0.2	7.8 ± 0.2	7.7 ± 0.2

Values represent mean ± SEM (*n* = 6).

**Table 2:** The effects of the *Ginkgo biloba* extract (10 mg/kg b.w., intraperitoneal) administered 24 h before titanium dioxide (5 mg/kg b.w., intravenous) on general parameters studied.

Parameter	5–24 h	24–48 h	48–72 h
Water consumption (mL in 19 or 24 h)			
Control	35 ± 3.4	32 ± 3.1	35 ± 1.9
Control Gb	23 ± 5.0	42 ± 7.2	32 ± 5.2
Treated TiO_2_	35 ± 5.1	32 ± 4.1	30 ± 4.1
Pretreated Gb	25 ± 3.3	38 ± 2.4	33 ± 2.2
Food consumption (g in 19 or 24 h)			
Control	18 ± 1.3	19 ± 0.7	21 ± 1.4
Control Gb	14 ± 1.5	14 ± 5.1	12 ± 4.1
Treated TiO_2_	19 ± 1.1	16 ± 1.3	15 ± 1.4
Pretreated Gb	18 ± 1.2	17 ± 1.4	17 ± 1.2
Body weight (g in 19 or 24 h)			
Control	305 ± 10	302 ± 10	299 ± 11
Control Gb	282 ± 24	280 ± 18	269 ± 23
Treated TiO_2_	227 ± 13	237 ± 14	246 ± 16
Pretreated Gb	278 ± 13	270 ± 16	273 ± 33

Values represent mean ± SEM (*n* = 6).
